# Sewage Protein Information Mining: Discovery of Large Biomolecules as Biomarkers of Population and Industrial Activities

**DOI:** 10.1021/acs.est.3c00535

**Published:** 2023-07-18

**Authors:** Montserrat Carrascal, Ester Sánchez-Jiménez, Jie Fang, Carlos Pérez-López, Antoni Ginebreda, Damià Barceló, Joaquin Abian

**Affiliations:** † Biological and Environmental Proteomics Group, Institute of Biomedical Research of Barcelona, Spanish National Research Council (IIBB-CSIC/IDIBAPS), Rosellón 161, E-08036 Barcelona, Spain; ‡ Department of Environmental Chemistry, Institute of Environmental Assessment and Water Studies (IDAEA-CSIC), Jordi Girona 18-26, 08034 Barcelona, Spain

**Keywords:** environmental proteomics, sewage epidemiology, water fingerprinting, mass spectrometry

## Abstract

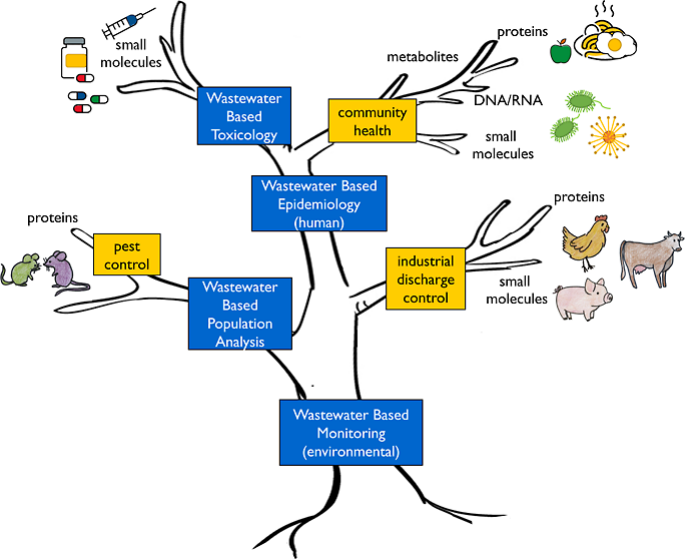

Wastewater-based epidemiology has been revealed as a powerful approach for surveying the health and lifestyle of a population. In this context, proteins have been proposed as potential biomarkers that complement the information provided by currently available methods. However, little is known about the range of molecular species and dynamics of proteins in wastewater and the information hidden in these protein profiles is still to be uncovered. In this study, we investigated the protein composition of wastewater from 10 municipalities in Catalonia with diverse populations and industrial activities at three different times of the year. The soluble fraction of this material was analyzed using liquid chromatography high-resolution tandem mass spectrometry using a shotgun proteomics approach. The complete proteomic profile, distribution among different organisms, and semiquantitative analysis of the main constituents are described. Excreta (urine and feces) from humans, and blood and other residues from livestock were identified as the two main protein sources. Our findings provide new insights into the characterization of wastewater proteomics that allow for the proposal of specific bioindicators for wastewater-based environmental monitoring. This includes human and animal population monitoring, most notably for rodent pest control (immunoglobulins (Igs) and amylases) and livestock processing industry monitoring (albumins).

## Abstract 2

The information carried by proteins in wastewater is still to be uncovered. A large-scale proteomics approach reveals the potential of these biomarkers for developing public health monitoring systems.

## 1 Introduction

Sewage chemical-information mining (SCIM),^1^ of which wastewater-based epidemiology (WBE), also known as sewage epidemiology, is the more relevant branch, has arisen as a complementary alternative to provide comprehensive health and environmental information on communities. Under this approach, sewage is regarded as an integrated pooled sample of the entire population served by a certain wastewater system; thus, its monitoring provides an average picture of its health status and activities.^1−3^

The success achieved through SCIM has been closely related to instrumental development, especially on mass spectrometry (MS) for the analysis of small and large molecules, and more recently by the introduction of techniques for the analysis of genetic material.^4^ Some successful applications of SCIM include the consumption of illegal drugs,^5,6^ pharmaceuticals and personal care products,^7,8^ tobacco^9^ and alcohol use,^10^ the exposure to toxicants like pesticides,^11^ and Bisphenol A,^12^ and with regard to biological response, oxidative stress^13^ or the monitoring of coronavirus prevalence during the recent COVID-19 outbreak.^14,15^

In this context, several authors have stressed the potential relevance of proteins in wastewater as health and environmental biomarkers.^2,4^ Early studies already evidenced the presence of enzymatic activity in the effluent of wastewater treatment plants (WWTPs),^16^ and human keratins and pancreatic elastase were identified among a few other bacterial proteins in sludge using the proteomic technology available at that moment.^17^ The presence of human proteins in sludge evidenced its resistance to degradation in wastewater and through the WWTP treatment and raised the question of their effect in the receiving waters.^16^ More recently, using ELISA analyses, quantitation of human immunoglobulins A and G in wastewater was reported and proposed as a tool for community serology.^18^ Besides these works, most sewage proteomic studies have focused on the characterization of the microbiome in either sludge^19^ or wastewater,^16,20^ and the information on other human, animal, or vegetal proteins remains scarce at best.

The current status of proteomics technologies allows sensitive and extensive analysis of very complex protein mixtures such those in wastewater. Disentangling the wastewater proteome would open the window to a new class of potential markers for SCIM purposes and would be the first step for developing new specific, targeted analytical methods to monitor anthropogenic activities and community health status in a non-intrusive way.

With this aim, in preliminary studies,^21,22^ we used passive sampling polymeric devices and liquid chromatography coupled to high-resolution MS shotgun proteomic methods, to expand, for the first time, the proteomic profiling of wastewater beyond prokaryotes to eukaryote higher organisms, covering plants, animals, and human proteomes. For the latter, we were able to identify not only the major proteome constituents, such as albumins and keratins, but also other less abundant proteins (for example, S100A8, uromodulin, and defensins), which are known as potential disease biomarkers. This seminal work can thus be regarded as a first attempt to disentangle the entire wastewater proteome, and, simultaneously, it highlighted the experimental and analytical challenges involved in its characterization.

In our previous work, the heterogeneity and complexity of the water samples drove us to use semisolid polymer probe in order to trap wastewater protein and allow their analysis minimizing interferences. While the method was effective, it requires letting the probe submerged for many days. Further, the set of proteins trapped was very probably biased by the polymer affinity or the formation of biofilms in their surface. Consequently, we focused on developing strategies for the characterization of the proteome directly from wastewater using existing automatic infrastructure for water collection at WWTP entrances. Here, we present our results on the characterization of the soluble fraction of the wastewater proteome (filtered through 200 nm pore) from 10 different municipalities in Catalonia covering a wide range of population sizes and influent characteristics (relative contribution of domestic and industrial load).

The objectives of the present study were: (a) the deep proteomic characterization of the wastewater soluble fraction and (b) to describe the observed protein pattern and their possible correlation with human activity in order to identify potential biomarkers that could be validated for new applications for SCIM or WBE or become monitoring targets for the improvement of WWTP operation and management.

Consequently, first we will describe the collection of proteins identified, their origin, distribution and possible correlation with anthropogenic activities, and then we will discuss the potential utility of some of the more abundant and ubiquitous human and animal proteins families identified in wastewater.

## 2 Material and Methods

Twenty-four-hour composite wastewater samples were collected at the inlets of 10 WWTPs in Catalonia (Figure S1, Table S6) in three different times of the year. These WWTP receive influents from different municipalities with populations ranging 28,000 (Banyoles) to 1,500,000 (Besòs) as well as diverse activities (Table S1). Samples were collected in the framework of the Catalonian Net for SARS-CoV-2 surveillance.^23^ Their collection, transport, and storage were made under standardized procedures before being processes for specific analyses.

For soluble proteins, 100 mL wastewater sample was centrifuged, filtered through 0.2 μm filters, lyophilized, redissolved in milli-Q water, and concentrated using a 10 kDa cutoff device. Samples were concentrated to approximately 400 μL. For proteins in the particulate, samples were ultracentrifuged twice with a wash step with phosphate-buffer. Then, pellets were lysed with beads as described by Casas et al.^24^

Soluble and particulate proteins were cleaned and concentrated in the heads of a sodium dodecyl sulfate-polyacrylamide gel electrophoresis gels. The bands of concentrated proteins were excised and digested with trypsin using an automatic device (DigestPro MS, Intavis), as previously described.^25^ For protein identification, tryptic digests were injected into the chromatographic system coupled to an Orbitrap-Velos High-Resolution Mass Spectrometer. Shotgun spectrometric analysis was performed in data-dependent mode^26^ and MS/MS spectra were searched using Protein Discoverer with the usual parameters for tryptic peptides^24^ and a 1% False-Discovery-Rate.^27^ The database used for searching was UniProt (rev. 08-22). The MS proteomics data have been deposited to the ProteomeXchange Consortium via the PRIDE^28^ partner repository with the dataset identifier PXD038781.

Regarding data treatment and comparative analysis, only proteins assigned as master proteins, with at least two peptides pointing to them, were considered for discussion. Estimation of the relative abundance of proteins was based on normalized spectral counts (NSCs). NSCs correspond to the total peptide sequence matches (PSM) obtained using Protein Discoverer and normalized to the mass of the protein to consider that the number of tryptic peptides produced by a protein increases with its size, and thus also the total PSMs measured. For the comparison of proteins such as amylases and albumins, we selected peptides with an unambiguous match to the protein (no other proteins in the Protein Group) and with at least two PSMs. Protein areas were calculated as the sum of all selected peptides pointing to the protein and were normalized to the wastewater flow measured at the WWTP inlet when the sample was collected. A more detailed material and methods is described in Supporting Information.

## 3 Results and Discussion

### 3.1 Wastewater Proteome

**Figure 1:**
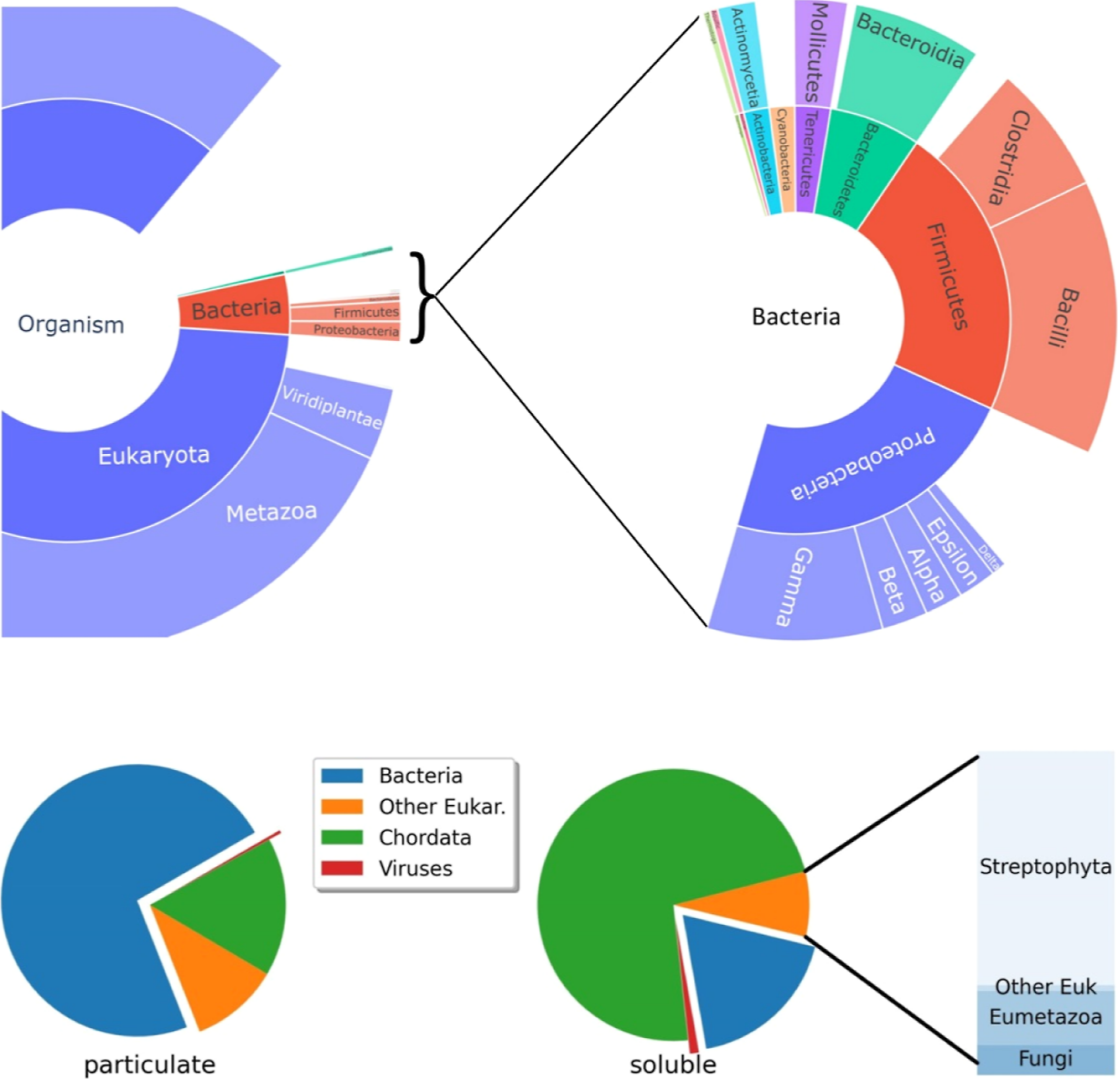
Distribution of Bacteria and Eukaryota proteins in the soluble fraction of wastewater and comparison with the particulate fraction (bottom). The sunburst graphs were prepared from the Unipept analysis of all peptides identified in the soluble fraction of wastewater samples. Comparison of particulate and soluble fractions was obtained from the multiconsensus analysis of all available samples, as described.

**Figure 2:**
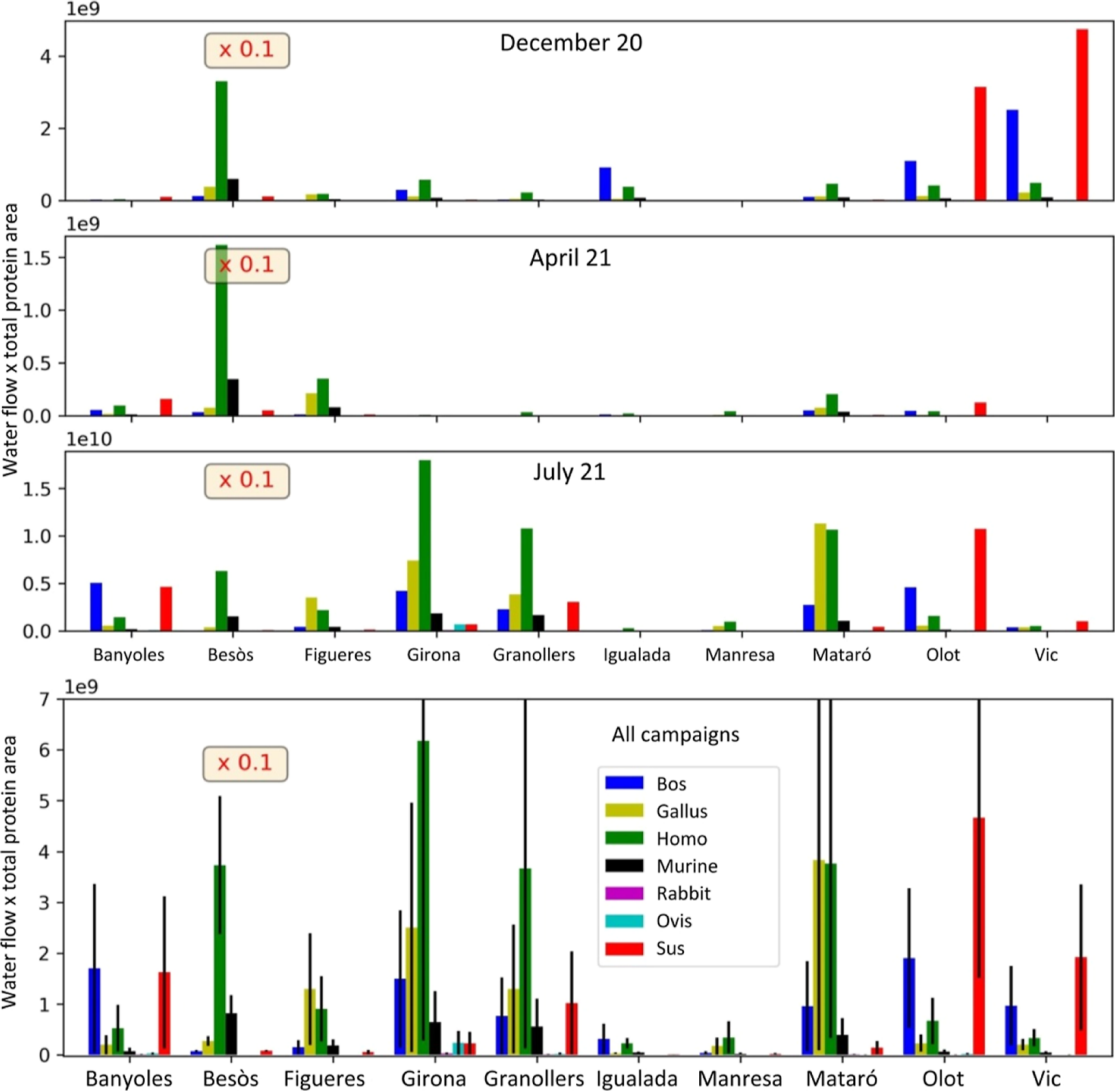
Distribution of proteins by species of origin in the different sampling sites by campaign (top) and with all campaigns combined (bottom).

**Figure 3:**
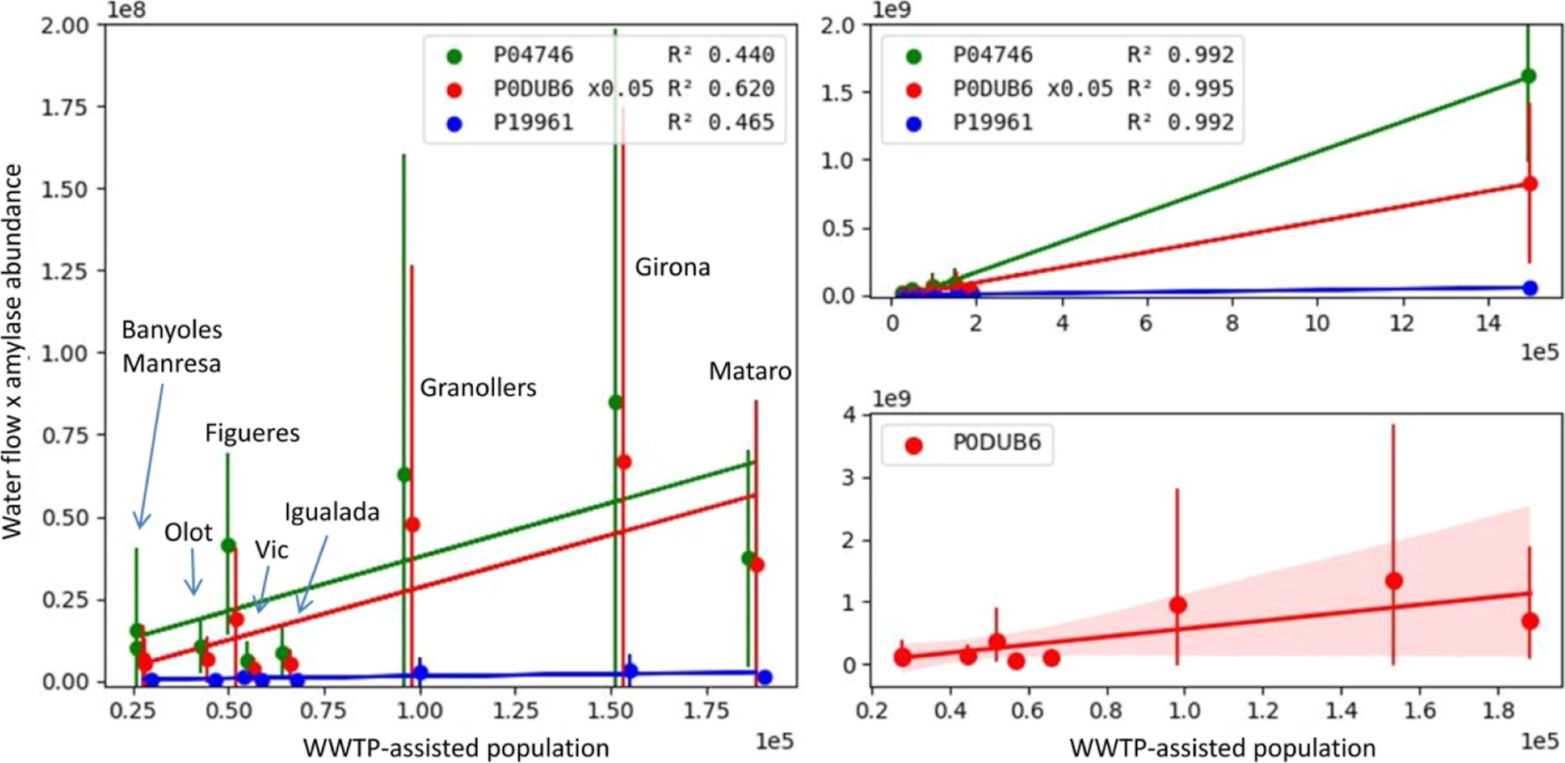
Human amylases represented versus the population assisted by the corresponding WWTP. Images include errors and regression lines obtained for the three amylases measured at all sites, except Besòs (left) and all sites (right, top), as well as the correlation for the major amylase (α-amylase 1A, P0DUB6) and the 95% confidence interval zone (Besòs not included) (right, bottom).

The non-targeted shotgun proteomics study of these water samples allowed us to identify a total of 4318 peptides (1% FDR, >1 PSM) that indicated 827 proteins (1% FDR, >1 peptide) (Table S4). The most abundant proteins were animal amylases and albumins (Table 1). Based on NSCs, eukaryotic proteins (mainly from mammals and birds) are the major components of wastewater, followed by bacterial proteins. Small amounts of viral proteins were also detected. Human proteins constituted 46% of the collection, followed by pig, chicken, cow, and rodent proteins (14, 9, 8, and 7%, respectively). Plant proteins made up >50% of all non-Chordata eukaryotes (Figures 1–3, Tables 1, and S4).

**Table 1:** 20 Most Abundant Proteins in the Wastewater Samples^a^^,^^b^

		entry name^c^					
accession	protein name	gene	species	coverage [%]	# peptides	# protein unique peptides	# NSCs
P04746	pancreatic α-amylase	AMYP	HUMAN	88	62	7	10924
P0DUB6	α-amylase 1A	AMY1A	HUMAN	86	58	9	10470
P19961	α-amylase 2B	AMY2B	HUMAN	88	60	1	9594
P08835	albumin	ALBU	PIG	93	119	91	8846
P01834	immunoglobulin κ constant	IGKC	HUMAN	93	13	2	7712
P01012	ovalbumin	OVAL	CHICK	75	32	22	7159
P02769	albumin	ALBU	BOVIN	92	118	70	5480
P01846	Ig λ chain C region	LAC	PIG	97	9	9	4773
P19121	albumin	ALBU	CHICK	88	85	84	4393
P02768	albumin	ALBU	HUMAN	90	87	14	4309
P83053	pancreatic α-amylase	AMYP	STRCA	33	22	2	3013
P00687	α-amylase 1	AMY1	MOUSE	23	15	2	2878
P0DOX7	immunoglobulin κ light chain	IGK	HUMAN	60	14	2	2786
P01009	α-1-antitrypsin	A1AT	HUMAN	60	35	17	2675
P00690	pancreatic α-amylase	AMYP	PIG	51	24	7	2627
P14639	albumin	ALBU	SHEEP	88	71	28	2540
P00689	pancreatic α-amylase	AMYP	RAT	28	14	2	2410
P07478	trypsin-2	TRY2	HUMAN	63	12	8	2283
P07724	albumin	ALBU	MOUSE	39	24	1	2173
P49064	albumin	ALBU	FELCA	32	26	5	2172
P09571	serotransferrin	TRFE	PIG	94	104	91	2159

a STRCA: ostrich; FELCA: cat.

b The complete list of identified proteins is available in Table S4.

c UniProtKB/Swiss-Prot entry name. The two terms of the entry name (gene_species) have been separated for convenience.

It is noteworthy that due to the remaining uncertainty in the spectrum-peptide matching process and the limitations of the protein inference approaches in shotgun proteomics, some artifactual assignments are expected in our protein collection, which has not been manually curated. This may be, for example, the case of the suspicious assignment of a pancreatic α-amylase protein (AMYP) to the common ostrich (*Struthio camelus*, STRCA) protein, as shown in Table 1. Although the assignment cannot be discarded a priori (there are ostrich farms in Catalonia), this is the case for a protein assigned on the basis of two forms of the same unique peptide sequence (oxidized and not oxidized) with a 94% identity with the human sequence. The probability of an incorrect assignment is reduced as the number of unique peptides pointing to a specific protein increases.

The most represented phylum in the collection was Chordata, and the major contributors to the proteins in this phylum were humans and livestock. Humans were represented by 243 proteins. The most abundant human proteins were pancreatic enzymes headed by α-amylases, making these proteins the main markers of human presence in wastewater. Several blood proteins (albumin, immunoglobulins [Igs], and complement proteins) and skin-derived proteins were also present in notable amounts. A DAVID gene ontology analysis^29^ revealed several enriched functional terms such as those related to the immune response (Igs, calprotectin, lactoferrin, lipocain, and dermcidin) or the anti-inflammatory response (meprin A, orosomucoid, and the serpin family). The most abundant non-human proteins detected in wastewater were albumin from cattle (and ovoalbumin from poultry). Albumin from commensal rodents (rats and mice) was one of the most important proteins detected.

Overall, our data showed two main sources of proteins in wastewater: excreta (urine and feces) from humans, and blood and other residues from livestock.

### 3.2 Wastewater Proteome is Compartmentalized

In an earlier study in which we used polymeric probes to capture proteins from wastewater, we found high levels of bacterial proteins in the samples.^21^ In contrast, in the filtered wastewater, bacterial proteins were relatively minor components, and the most abundant proteins differed from those found in the probes (Figures 1 and S2).

These differences can be explained by the formation of biofilms in the probes that become enriched in bacterial proteins. In addition, the fact that the samples were passed through a 0.2 μm filter suggests that most of the bacterial protein mass was transported in bacterial cells. A preliminary analysis of the particulate fraction of the wastewater samples confirmed this finding. We analyzed material from two different WWTP sites: a major urban area (Besòs) and a rural community (Vic). In both cases, bacterial proteins were the major components (Figure 1, bottom), although the distribution of the species was different. Although the bacteria-eukaryote distribution in the wastewater particulate was found to be similar to that of the polymeric probes, these two fractions showed some differences in the dominant proteins found in each of them (Figure S2, insert). A more in-depth study is required to confirm whether these differences are due to the potential selectivity of the polymeric probes or simply reflect the different origins of these samples.

Another interesting example of protein compartmentalization is human elastase 3A (CL3A), a protein that we are considering as a potential biomarker for the human population. Human elastase is a well-known component of sewage and WWTP sludge.^19^ This is a recalcitrant protein with a high concentration in feces,^16^ which we have described as the major component retained in our polymer probes. In contrast to most other Chordata proteins, which were located preferentially on the filtrates, CL3A was found in higher relative amounts in the particulate fraction, where it was the major component (second position in the particulates from Vic and Besòs). Other proteins found in large amounts, mostly in particulate fractions, were keratins. Whereas EFTU and 60 kDa heat shock proteins (CH60) would be the most abundant and pervasive markers of bacterial presence, for mammals this position would be occupied by keratins.

Although we aimed to describe the characteristics of filtrated wastewater in this work, these preliminary results on the particulate fraction reveal that this is only a partial view of the full wastewater proteome. Comprehensive and in-depth analysis of this proteome is, however, complicated. Our attempts to directly analyze the full wastewater composition (without the previous separation of the soluble and particulate fractions) were unsuccessful, likely due in part to inefficient trypsin digestion, probably caused by the interference of other compounds in the water. Therefore, we believe that parallel, separate analyses of the different wastewater compartments using sample fractionation methods, as those described here, may be the best strategy for a complete description of the wastewater proteomes. Knowledge of the protein distribution between these two compartments will further aid in the future development of methods to monitor potential biomarkers.

### 3.3 Semiquantitative Analysis of the Wastewater Proteome Characterizes the Human Activity Around the WWTPs

Wastewater collected from a community reflects its population and domestic and industrial activities.^1,2^ To test the potential of the wastewater proteome as a potential biomarker, we performed a comparative analysis of the protein composition at different collection sites and estimated the protein abundances considering the corresponding wastewater inflows.

For this purpose, protein semiquantitative data were calculated from the sum of the areas of their unique peptides in the ion chromatograms. Only those proteins unambiguously assigned by the search program (at least with a unique peptide pointing to it and with no other homologous members in the protein group) were considered. This procedure causes a bias in the set of proteins represented because those highly conserved between species produce only a few unique peptides or none in the worst case. Thus, highly conserved proteins are at risk of not being included in the set of quantified proteins. In return, this strict filtering prevents the contribution of peptides from similar proteins to the area of the one measured. As discussed previously, incorrect species assignment cannot be discarded for less represented species. This may be the case for the two proteins assigned to *Pongo abelii*, a species with high homology to humans, or the assignments to *Danio rerio* and *Dictyostelium discoideum*, two species that frequently appear in proteomic searches because of the assignment of proteins from other related species, but with genomes that have not been annotated to the same degree as those of these model species.

Using this approach, we obtained reliable quantitative data from a set of 489 proteins (between 350 and 401 proteins could be quantified in the different samples), representing a total of 112 species (26 from more than one protein [Table S2]).

The distribution of the major proteins in the different campaigns was highly variable (Figure 2). However, on average, they showed some characteristic traits that may indicate a relationship with the human population and industrial farming activities at each site, especially on the distribution profiles of pig, cow, and chicken proteins. A preliminary analysis of the data led us to select some protein groups (amylases, albumins, and Igs), with quantitative profiles that suggested that they could be potential biomarkers for further studies.

### 3.4 Amylases as Mammal Population Indicators

The most abundant protein in the wastewater soluble fraction was human pancreatic α-amylase. Rodent, pig, and chicken amylases were detected in lower amounts. Amylases are the major protein components in feces, together with elastases, and are present in minor proportions in urine.^30,31^ Pancreatic amylases and elastases are secreted in the pancreatic juice together with other lipases, nucleases, and proteases for the digestion of food. The main role of pancreatic enzymes in the intestine reflects their high stability against hydrolytic degradation. An example is pancreatic elastase, which has been detected in WWTP sludge, evidencing its resistance to the wastewater environment and WWTP treatment. In blood and sera, α-amylase maintains unaltered its enzymatic activity for at least a week at room temperature,^32^ so that protein sequence information can be expected to be preserved still longer. Thus, due to their high abundance in wastewater, their probable resistance to protease action and the species-specific information carried in their sequences, amylases may be potential markers of human population and, as such, a potential tool to normalize the abundances of other biomarkers. One important uncertainty in WBE studies is the estimation of the number of inhabitants served by a WWTP.^33−35^ The wastewater physicochemical parameters frequently used for this purpose [chemical oxygen demand (COD), biological oxygen demand (BOD), total nitrogen or phosphorous] are highly unspecific and census does not reflect population dynamics and is often outdated.^34^ In recent years, molecules excreted in human feces or urine have been evaluated and used as markers of population. Most of them are small molecules, such as creatinine, cholesterol, 5-hydroxyindoleacetic acid, caffeine, prostanol, or drugs widely used by the population. Human-specific protein forms such as amylases have the advantage of being virtually free of the contribution from other non-controlled exogenous sources and thus to provide more accurate measurements.

Amylase presence in wastewater greatly increased when comparing cities with large populations (Barcelona, Besòs WWTP) with small- and medium-sized cities (Figure 3, top-right). However, although a trend was observed between the amylase levels and the estimated population served by the WWTPs, the correlation was poor (Figure 3, left and bottom-right). Unfortunately, we did not have data from WWTPs serving populations in the middle range between Mataró and Besòs to obtain a more precise view.

The degree of correlation between population and amylase levels may be affected by inaccuracies in the population data. The population data used correspond to the available official figures (Table S1). However, this parameter is not exempt from uncertainty, as the actual population may be subjected to large fluctuations over time (for example, seasonal tourism) or may not reflect the actual population channeling their sewage into the WWTP. Another factor to consider is the robustness of the biomarker as different protein degradation dynamics in the specific biological and physicochemical environments of the different zones could affect the measured levels of amylase in water. Further research is required to deconvolute these factors.

Since factors like changes in water flow due to precipitations that affect the levels of amylases in wastewater would be common to other proteins in wastewater, amylases could be useful to correct for these factors the amount of other human protein biomarkers or the amount of these pancreatic enzymes from other species. A potential application of great interest would be their use to monitor rodent populations in urban areas. Rat pests are a human health hazard because of the diseases they can transmit through the bacteria that infect them, and the transmission of fleas, ticks, and mites. In addition, they threaten the integrity of infested structures, and once established, their elimination is difficult. In large cities, rats live in the sewers. If no control action is taken, these animals can live between 2 and 3 years and procreate up to five times a year with an average of 4–8 offspring; thus, their number varies rapidly over a few months.^36^ Various strategies are currently being used to monitor these pests, generally based on animal counts and extrapolation to the total population.^37^ The number of animals in a large city is often referred to as the number of rodents per inhabitant. For example, it is estimated that in the city of Barcelona, there may be one rat per eight inhabitants,^38^ and some estimates speak of up to 0.25 per inhabitant in the city of New York.^39^ However, there is no standardized method to determine their numbers, estimate population density, or understand population dynamics.

**Figure 4:**
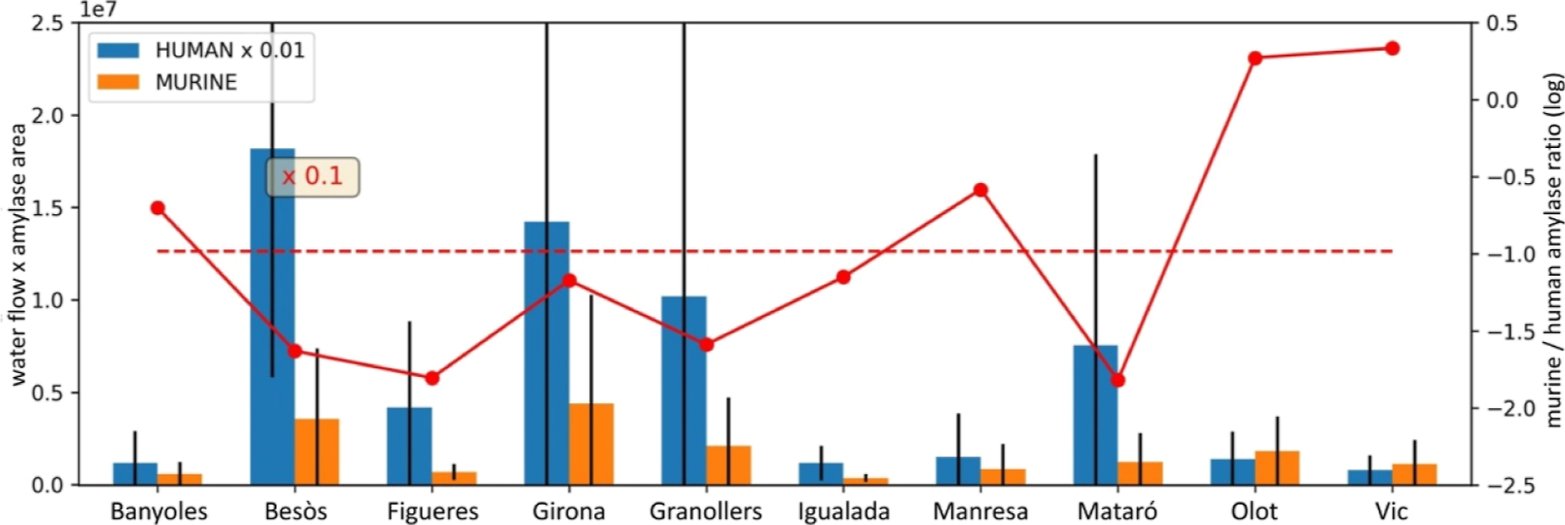
Murine (rat and mouse) and human amylases in the different sites. The red line indicates the murine-to-human ratio, and the red-dashed line marks the mean ratio.

As in humans, rat amylases are secreted into the pancreas and excreted mainly in feces; therefore, their quantification in wastewater relative to human amylases may allow the detection of rodent population peaks. Murine amylases were found in water in 100–500-fold lower amounts than human amylases. The ratio of murine to human amylases varies with the site and is generally higher in small cities in predominantly rural areas (Banyoles, Olot, and Vic) and smaller in large urban industrialized areas (Besòs/Barcelona, Mataró) (Figure 4). Interestingly, a peak in the murine-to-human amylase ratio was observed in the July campaign in Igualada. This sample also showed a marked increase in the rat-to-mouse amylase ratio despite the fact that in all other samples the ratio remained relatively constant (Figure S7). Whether this could reflect an increase in the rat population at this site is difficult to determine from a single event but these preliminary findings have prompted us to conduct additional studies that are now underway for this particular application.

Currently, work is underway to develop a targeted-MS method that allows a more precise quantification of human and rodent amylases in wastewater. Knowledge of the best tracking subjects (those more abundant, unique, and species-specific peptides) should facilitate future development of immunoaffinity-based sensors for this purpose. The validation of this approach would be the source of new tools for pest surveillance that can provide integrated information on the area of origin and conduction of the waste in parallel with other more local methods already in use (photo trapping, rat traps...).

### 3.5 Albumins as Livestock Industry Markers

Albumins were found in high quantities in the analyzed water samples. The presence of albumin in wastewater probably results from industrial discharge of animal blood. Serum albumins are 60–70 kDa proteins with a high sequence homology among many primates (>90% identity) and other mammals (>50%). Differences between homologous albumins are widely distributed throughout their chains, resulting in significantly different sets of tryptic peptides after enzymatic digestion. Thus, considering albumins from humans, livestock, poultry, common human pets (cat and dog), and murids (rat and mouse), there are always between 24 and 42 different peptides potentially identifiable by our proteomics approach (>6 AA), which are unique to any pair of these species (Figure S3). Considering the full set, any of these albumins would theoretically yield between 21 and 38 unique canonical tryptic peptides, which can allow species-specific identification and quantification.

Feces and blood disposed of by slaughterhouses are of great concern as water pollutants.^40,41^ Albumin is the largest protein component in sera (approximately 50–60% weight in humans). This high abundance and the differences in albumin sequences between species open the possibility of developing MS-based strategies for specific monitoring of the levels and sources of biological contamination downstream of the release point and at the WWTP. This could be a powerful monitoring tool not only for environmental studies assessing the status of a water body but also for regulatory agencies in the surveillance of controlled and uncontrolled discharges of animal residues in rivers and wastewater systems. Currently, occasional discharge can be indirectly detected by routine monitoring of the organic load content in wastewater (for example, BOD5, COD, and TOC); however, these methods do not provide information on the contributing molecules or their origin.^41^

In our study, we identified between 1 and 84 unique peptides for the different albumins considered above, which are, in some cases, higher than the expected canonical tryptic sequences because they also include peptides with missed cleavages (incompletely digested peptides). The number of unique peptides detected was highly dependent on the concentration of a specific protein in the sample. Thus, pig albumin is more representative, whereas no unique mouse peptide passed the data treatment quality filters (Figure S3).

As albumins are the major contributors to animal protein mass in the wastewater proteome, the albumin profile distribution was highly similar to that shown for the total protein distribution (Figure 2). In concordance, the profiles of farm animal albumins were found to be significantly different among the sites (Figure S4). To determine the correlation between these albumins and the presence of the corresponding species at a given site, we compared the number of official livestock units in each area with measured albumin values (Figure S5). We found that livestock units and albumin abundance were significantly different from each other; for example, wastewater samples from areas with a relevant number of pig farms, such as Figueres, Igualada, and Manresa, contained significantly low amounts of pig albumin. As animal blood and tissues are the major containers of albumins, these proteins probably mark the presence in the sewage of animal residues from the meat industry (for example, slaughterhouses), whereas livestock units reflect the number of animals raised in the region. For example, in Mataró, where the most important Spanish company in the poultry processing sector is located, there is a significant difference between the poultry livestock units and the measured Gallus albumin levels. Similarly, pig albumin appears to be the main albumin in Vic, Olot, and Banyoles, cities in which the pork industry is of great importance.

### 3.6 Human Immunoglobulins

Another important family of proteins found in samples is human Igs. Recently, human Igs were proposed as health biomarkers, although their presence, distribution, and detectability in wastewater had not been assessed.^15^ More recently, measurement of specific Igs in wastewater was proposed as a window for community serology and an ELISA method was developed in the context of COVID surveillance.^18^ Igs are large heterodimeric glycoproteins composed of two heavy and two light chains, each of which is a combination of different variable and constant domains encoded by 176 genes. There are five Ig isotypes named based on their α, Δ, ϵ, γ, and μ heavy chains (IgA, IgD, IgE, IgG, and IgM, respectively), each containing one of the two classes of light chains (κ and λ). Both heavy and light chains were subdivided into highly homologous subtypes, each with a different entry in the UniProt database. This greatly complicates proteomic quantitation by measuring the areas of unique peptides. Thus, many of the Ig tryptic sequences identified in our samples indicated two or more different Ig sequence accessions; consequently, they were not selected for measurement. This led to a situation where we had no unique peptides to quantify the λ chain, or where the areas of the γ chain, which makes the major Ig in the blood, were relatively small and unreliable, as were calculated from a minor unique peptide. As multiple protein assignations of the identified peptides were always to subtypes of the same chain, we measured each Ig chain, considering all its subtypes together. This enabled us to measure three heavy chains and two light chains (Figure S6). Sequences pointing to the J-chain, a component of IgA and IgM, and variable sections of the heavy chains not related to a specific Ig were also measured.

The Ig chain profiles were similar among the different sites and through the different campaigns (not shown), whereas the areas varied greatly, likely correlating with the human population served by the corresponding WWTP site. As observed in the profiles for other proteins, Ig abundance changed significantly between campaigns, as reflected in the length of the corresponding error bars (Figure S6). Girona showed the highest difference, with an 8105-fold change between two campaigns, whereas Besòs (Barcelona) had the lowest maximum difference, with a fivefold change. As in the case of amylases, these variations could be partly a reflection of changes in the population at the site. This hypothesis is supported by the profiles of the human-Igs-to-amylases ratio, which, by comparison, were relatively constant between sites and campaigns (Figure S6, right). Thus, the greatest variation in the Igs/amylase ratio was 11-fold (Mataró), and the smallest maximum difference was only 1.4-fold (Besòs). On average, a 932 ± 2396 Ig maximum fold change was calculated between campaigns, whereas this average was 4 ± 3-fold for the Ig/amylase ratio, >2.5 orders of magnitude lower. These results further support the interest of abundant human proteins such amylases to normalize the abundance of other human proteins in wastewater.

In summary, our shotgun analysis reveals the high abundance of antibody molecules in wastewater, and the capability to discern between different Ig types and chains as well as to determine their profile, thus providing new knowledge for further development on SCIM methods based in these molecules.

### 3.7 Wastewater Origin can be Differentiated by a Small Group of Biomarkers

**Figure 5:**
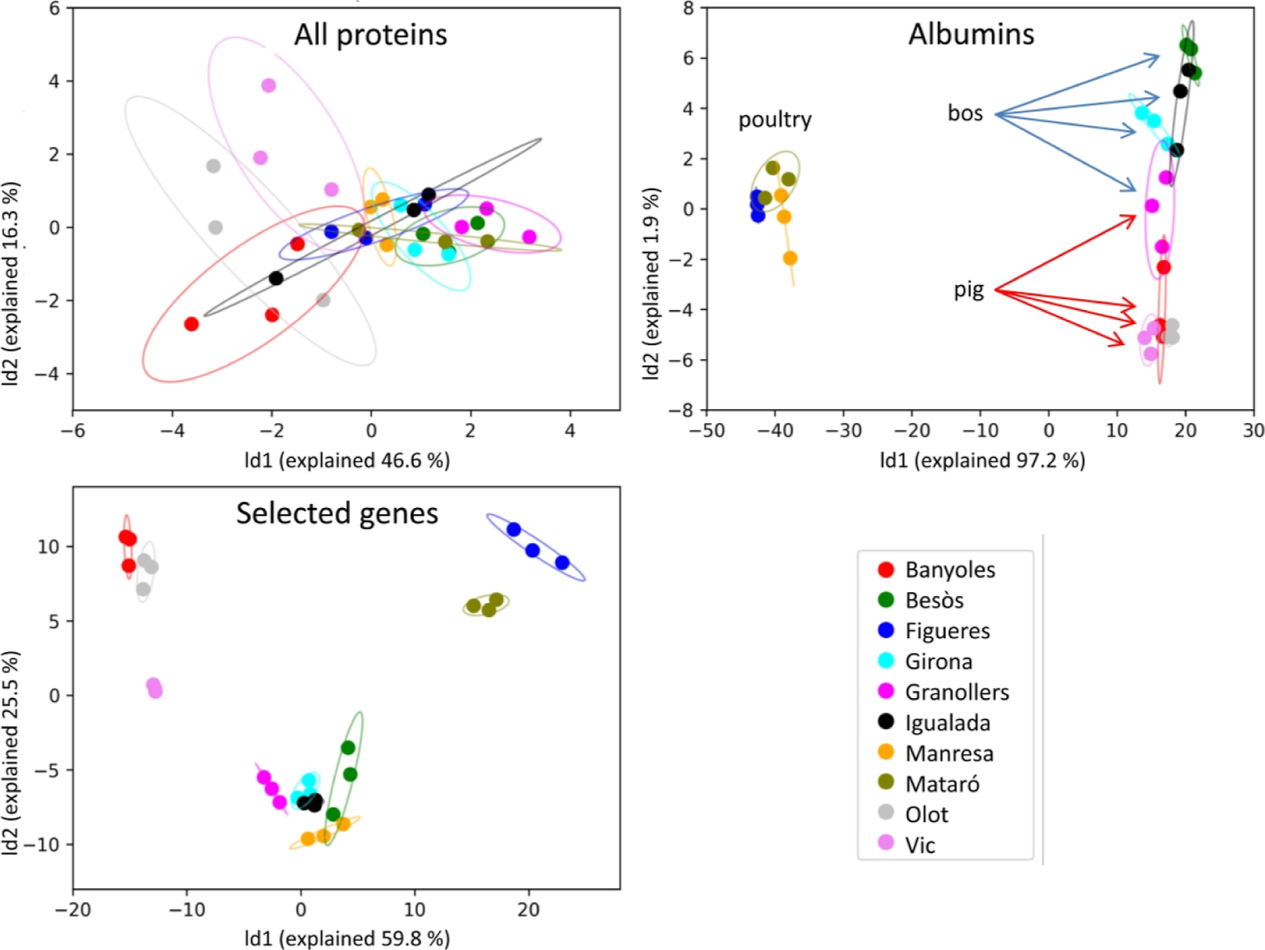
LDA of the full proteome profiles (top-left), the albumin profiles (top right), and of a pre-selected group of protein markers (bottom).

We have shown that wastewater proteomes exhibit protein profiles that are characteristic of the site and time frame. Protein profiles contain information on human activities in a given area, as revealed when considering livestock activities. This opens a window for monitoring diverse activities or site statuses through the determination of these protein patterns. Based on these premises, we tested the possibility of automatically differentiating wastewater origin by employing its proteome profile. For this purpose, we performed different classification and clustering analyses, which produced poor outputs (Figure 5, top left). Wastewater is a highly complex matrix, and many of its components contribute little to distinguishing features that facilitate discerning between samples. Instead, they introduce noise, acting as confounding factors in the classification. In contrast, a linear discriminant analysis (LDA) using the albumin subproteome showed clear differentiation over the first component of the sites with dominant poultry farming (Figueres, Mataró, and Manresa) from the others. The latter, in turn, are distributed along the second component, differentiating those with a predominance of cattle from those with a predominance of pigs (Figure 5, top-right).

To optimize the classification, we used a protein set derived from the genes represented by the proteins with higher loads in discriminant factor analysis. This set was composed of 24 proteins expressed by ALB, SERPINB14, SERPINA1, SERPINA3-1, AMY1A, Amy2, TF, and Alpi genes in humans and other animals (Table S3). Analysis of this set produced a significantly more resolved separation of different clusters (Figure 5, bottom).

In summary, the present study greatly extends the knowledge we achieved in the comprehensive characterization of the wastewater proteome reported in our preliminary research.^21^ Here, we focused on the potential relevance of these protein profiles as new SCIM tools. To this end, analytical methods based on LC-HRMSMS shotgun proteomics were developed for both the dissolved and particulate materials contained in wastewater samples collected at the inlet of 10 WWTP serving municipalities in a broad range of population sizes.

Our data provide a comprehensive description of wastewater proteins, their distribution among different organisms, and a semiquantitative analysis of many of them. The data presented encompassed both prokaryotic (bacteria and, to a lesser extent, viruses), non-Chordata (plants), and Chordata eukaryotic organisms (including birds, mammals, and humans), which notably expands the scope of previous studies performed in wastewater and sludge, specifically focusing on the bacterial proteome.^16,20^

We describe two main differential sources of proteins: excreta (urine and feces) from humans, and blood and other residues from livestock. The results highlighted significant differences between the proteomes in the soluble (filtered) phase and the particulate material, dominated by Chordata and bacterial proteins, respectively. Our findings also provide new insights into the wastewater proteome that allow pointing out the possible practical use of some potential bioindicators in relation to wastewater-based environmental monitoring and WWTP management. Some relevant examples include amylases for mammalian population monitoring (applicable, for instance, to rodent pest surveys) and albumins as indicators of the cattle processing industry. Finally, in our previous work, we noted the presence in wastewater of endogenous human molecules, which are known disease biomarkers. Although we did not focus on human epidemiology, this study provides useful additional information on the presence of these and other endogenous human molecules of possible interest for WBE. The requirements that a protein must meet to be used as biomarker in WBE have been discussed in depth elsewhere^2^ including a well-defined disease–biomarker correlation, their excretion in high amounts and their stability both in vivo and in the wastewater media. Currently, the number of potential candidates is still small and none has been demonstrated yet.^2^ Some limitations of the proteomics approach such as the need for specialized equipment and trained personnel have likely contributed to the situation. However, we can hope that once a potential biomarker is deemed worthy of further investigation, methods other than MS can be used for further validation and large-scale application.

Collectively, our prospection of the wastewater proteome is far from complete and raises new, unexpected scientific questions about the observed protein profiles. This is a consequence of our still limited knowledge about the numerous factors involved in protein dynamics along their route from the emission site to the sampling site as well as the actual emission rates of these proteins over time. Still, the enormous potential of proteins as health and environmental biomarkers compels to an exhaustive characterization of possible confounding factors in order to develop accurate, robust applications for these molecules.

In conclusion, we have demonstrated for the first time the feasibility of wastewater proteome mining using modern proteomic technologies and have provided a protein database of value for future SCIM studies. We have shown that proteins in wastewater carry unique and specific information about their origin and we anticipate that these characteristics will open new avenues for the future development of new applications for environmental surveillance and monitoring.

## Supporting Information Available

Location of sampling site; distribution of proteins between soluble and particulate fractions; albumin sequence differences between species; albumin profiles from farm animals; comparison of protein data with livestock units; human immunoglobulins in wastewater; rat and mouse amylases; WWTPs working data; species quantified; and site discriminant proteins (PDF)
Peptides and proteins identified in the soluble wastewater proteome (XLSX)
Proteins identified in the soluble, particulate and in the previous analyzed probes (XLSX)
WWTP and physicochemical characteristics of the wastewater samples (XLSX)

## Abbreviations

SCIM: sewage chemical-information mining
WBE: wastewater-based epidemiology
MS: mass spectrometry
LC-HRMS/MS: liquid chromatography coupled to high-resolution tandem mass spectrometry
BSA: bovine serum albumin
WWTP: wastewater treatment plant
SDS-PAGE: sodium dodecyl sulfate-polyacrylamide gel electrophoresis
NSC: normalized spectral count
PSM: peptide sequence match
Ig: immunoglobulin
TFA: trifluoroacetic acid
PCR: polymerase chain reaction
RT-PCR: reverse transcription polymerase chain reaction
NMWL: nominal molecular weight limit
GO: gene ontology
