# Supplementary material for: Sewage Protein Information Mining: Discovery of Large Biomolecules as Biomarkers of Population and Industrial Activities
Source: Environ Sci Technol. 2023 Jul 18;57(30):10929–39. doi: 10.1021/acs.est.3c00535 (PMC10399289; doi:10.1021/acs.est.3c00535)
Supplement: Supplementary file 1 — es3c00535_si_001.pdf [file es3c00535_si_001.pdf]

Sewage Protein-Information Mining: Discovery of Large Biomolecules as Biomarkers of Population and Industrial Activities

Montserrat Carrascal[a], Ester Sánchez-Jiménez[a,b], Jie Fang[a,b], Carlos Pérez-López[a,b], Antoni Ginebreda[b], Damià Barceló[b,c] and Joaquin Abian[a*]

[a] Biological and Environmental Proteomics, Institute of Biomedical Research of Barcelona, Spanish National Research Council (IIBB-CSIC/IDIBAPS), Rosellón 161, E-08036 Barcelona, Spain

[b] Institute of Environmental Assessment and Water Studies (IDAEA-CSIC), Department of Environmental Chemistry, Jordi Girona 18-26, 08034 Barcelona, Spain

[c] Catalan Institute for Water Research (ICRA), Emili Grahit 101, Parc Científic i Tecnològic de la Universitat de Girona, Edifici H2O, 17003 Girona, Spain

Contents

Material and methods ………………………………………………………….. S2 - S5

Figures S1 to S6 ………………………………………………………….. S6 - S11

Tables S1 to S3 ………………………………………………………….. S12 - S14

Tables S4 to S6: individual excel files.

MATERIAL AND METHODS

1. Sample collection

Twenty-four-hour composite wastewater samples were collected at the inlets of 10 wastewater treatment plants (WWTPs) located in the Girona and Barcelona provinces in Catalonia (Supplementary Figure 1). An automatic water sampler was used at all sites. The samples were then transferred to the laboratory at 4 °C.

Three collection campaigns were conducted on the 14th of December 2020, and on the 19th of April and 26th of July 2021 (winter, spring, and summer campaigns, respectively). For the study of the particulate fraction, samples were collected on three different days at the entrance of WWTP Besòs and Vic in May 2022.

Data on water inflow measured on the day of collection were provided by the WWTP operators.

2. Sample preparation

Soluble Proteins. The collected samples were filtered immediately after arrival at the laboratory. For this, up to 100 mL of 24-h composite wastewater sample was centrifuged at 4000 × g (10 °C, 20 min), and the supernatant was filtered through 0.2-μm filters (VWR, North American, USA). The filtered samples were lyophilized using a freeze-dryer (TELSTAR LyoAlfa 6, PA, USA).

For the analysis, lyophilized samples were reconstituted in 20 mL MilliQ water and concentrated using a 10 kDa cutoff device (Amicon®, NMWL 10 kDa), with a filter that was previously passivated to minimize protein adsorption. Passivation was performed by washing the filter with 2.4 mL of NaOH 0.1M that was eliminated by centrifugation at 4000 × g (13 °C, 10 min), followed by a second wash with 2.5 mL milliQ water and centrifugation. Finally, filters were immersed overnight in Tween-20 (5% in MilliQ water), extensively washed with MilliQ water, and centrifuged at 4000 × g (13 °C, 5 min). Samples were concentrated to approximately 400 μL, then evaporated to dryness using a SpeedVac. Proteins in the sample were cleaned and concentrated in the heads of sodium dodecyl sulfate-polyacrylamide gel electrophoresis (SDS-PAGE) gels (5% stacking and 12% resolving) at 50 V for 40–50 min. Bovine serum albumin (BSA) was used as a reference marker. After electrophoresis, the gels were stained with Coomassie Blue and scanned. The bands of concentrated proteins were excised and

digested with trypsin using an automatic device (DigestPro MS, Intavis), as previously described [25].

Proteins in Particulate. The three 24-h composite wastewater samples from each WWTP were combined, and up to 30 mL was processed. First, samples were centrifuged at 600 × g (15 °C, 15 min), and the supernatants were ultracentrifuged at 112700 × g (4 °C, 20 h, accel=9, desel=9) (Sorvall Discovery 90SE with rotor SW-28). Thereafter, the pellets were washed with phospahate-buffered saline using ultracentrifugation under the same conditions as described before.

After washing, the pellets were lysed with beads as described by Casas et al. [26]. Briefly, pellets were reconstituted in 500 µL of denaturing lysis buffer containing 4% SDS, 0.1 M DTT, and 100 mM Tris-HCl pH 7.5 through sonication in a bath (Ultrasons, J.P. Selecta) and incubated in a Thermomixer (Eppendorf, model F2.0) at 95 °9C (800 rpm, 1 h). Samples were then homogenized in a Bullet Blender (Next Advance Storm, NY, USA) for 3 min at speed level 8 using 250 µL zirconium silicate beads (0.1 mm diameter, BioSpec, 11079101z). After homogenization, the beads were pelleted by centrifugation at 18000 × g (10 min) and pellet lysates were recovered from the supernatant.

Approximately 100 µL of each sample (25% of the total) was concentrated using SDS-PAGE gels (5% stacking and 12% resolving) at 50 V for 40 – 50 min. BSA was used as a reference marker. After electrophoresis, the gels were stained with Coomassie Blue and scanned. The bands with concentrated proteins were excised and digested with trypsin using an automatic device (DigestPro MS, Intavis), as previously described [25].

3. LC-HRMS/MS and database search

The LC-HRMS/MS system consisted of an Agilent 1200 Series Gradient HPLC (consisting of a capillary nanopump, binary pump, thermostatic micro injector, and micro-switch valve) coupled to an Orbitrap-Velos High-Resolution Mass Spectrometer (ThermoFisher) equipped with a nanoESI ion source.

For the analysis, the tryptic digests of the sample extracts were evaporated until dry and re-dissolved in 50 µL of 0.5% TFA 5% methanol with gentle agitation in a Thermomixer (5 min, at 22 °C, 900 rpm). Five microliters of this solution was injected into the HPLC system.

Separation was performed on a 15-cm long, 100 µm i.d. C18 column (Nikkyo Technos Co.) preceded by a C18 preconcentration cartridge (Agilent Technologies). Separation was done at 0.4 µL/min using a 120-min gradient from 3–35% solvent B (solvent A: 0.1% formic acid, solvent B: acetonitrile 0.1% formic acid).

The Orbitrap-Velos was operated in positive ion mode with a spray voltage of 1.7 kV. Spectrometric analysis was performed in data-dependent mode, acquiring a full scan followed by 10 MS/MS scans of the 10 most intense signals detected in the MS scan. Full MS (range 400-1650) was acquired in the Orbitrap with a resolution of 60.000. MS/MS spectra were obtained in a linear ion trap.

MS/MS spectra were searched using SEQUEST (Proteome Discoverer v2.5, ThermoFisher) with the following parameters: peptide mass tolerance, 20 ppm; fragment tolerance, 0.8 Da; enzyme, trypsin, and allowance of up to two missed cleavages; dynamic modification, methionine oxidation (+16 Da); and fixed modification, cysteine carbamidomethylation (+57 Da). Searches were performed using UniProt (rev. 10-21). Final results were filtered using peptide rank 1, peptide confidence high (0.1% FDR), and search engine rank 1.

The MS analysis of the particulate samples was performed as described above, except for the use of a different chromatographic system that consisted of a C18 column trap (nanoEase^{TM} M/Z Symmetry C18 100 Å, 5 µm, 180 µm × 20 mm, Waters Corporation) connected to a 25 cm long, 75 µm i.d. C18 column (nanoEase^{TM} M/Z HSS C18 T3 100 Å, 1.8 µm, 75 µm × 250 mm, Waters Corporation). The separation was done at 0.4 µL/min in a 180-min gradient from 2–40% solvent B (solvent A: 0.1% formic acid, solvent B: acetonitrile 0.1% formic acid). The HPLC system was composed of a µBinary Solvent Manager, µSample Manager, and Trap Valve Manager from the Acquity UPLC M Class (Waters Corporation).

MS/MS spectra of the particulate extracts were searched using SEQUEST (Proteome Discoverer v1.4, Thermo Fisher) software with the same parameters as above. The search included a reanalysis of the MS data from the soluble extracts and extracts from the material found in the polymeric probes used in our previous work [23]. The database used for searching was UniProt (rev. 08-22). The mass spectrometry proteomics data have been deposited to the ProteomeXchange Consortium via the PRIDE [27] partner repository with the dataset identifier PXD038781.

5. Data treatment and semiquantitative analysis

Overall descriptions of the soluble wastewater proteome were obtained from the protein identification output of a Protein Discoverer Multiconsensus analysis, including all protein identifications from the different sites and campaigns. For discussion purposes, only proteins assigned as master proteins, with at least two peptides pointing to them, were considered. Estimation of the relative abundance of proteins was based on normalized spectral counts (NSCs). NSCs correspond to the total peptide sequence matches (PSM) obtained using Protein Discoverer and normalized to the mass of the protein to consider that the number of tryptic peptides produced by a protein increases with its size, and thus also the total PSMs measured.

The comparison of the soluble and particulate proteomes of the material in this study and that of the material found in the polymeric probes [23] was performed as described above. They included all soluble extracts, two replicates of particulate samples from the Vic and Besòs WWTPs, and all the probe-derived sample analyses from the inlet of the WWTPs (site 1 of the three WWTP samples in our previous work). Owing to the high number of archives to process, multiconsensus protein identification was performed considering five data groups: soluble data combined by campaign, combined particulate data, and combined probe data.

For the semiquantitative determination of proteins such as amylases and albumins, we selected peptides with an unambiguous match to the protein (no other proteins in the Protein Group) and with at least two PSMs. Unselected peptides pointing to a protein in the unambiguous set were then recovered and added to that set. Protein areas were calculated as the sum of all selected peptides pointing to the protein and were normalized to the wastewater flow measured at the WWTP inlet when the sample was collected.

To ensure reproducibility and traceability, Protein Discoverer output data were processed and documented using Jupyter notebooks and Python.

FIGURES

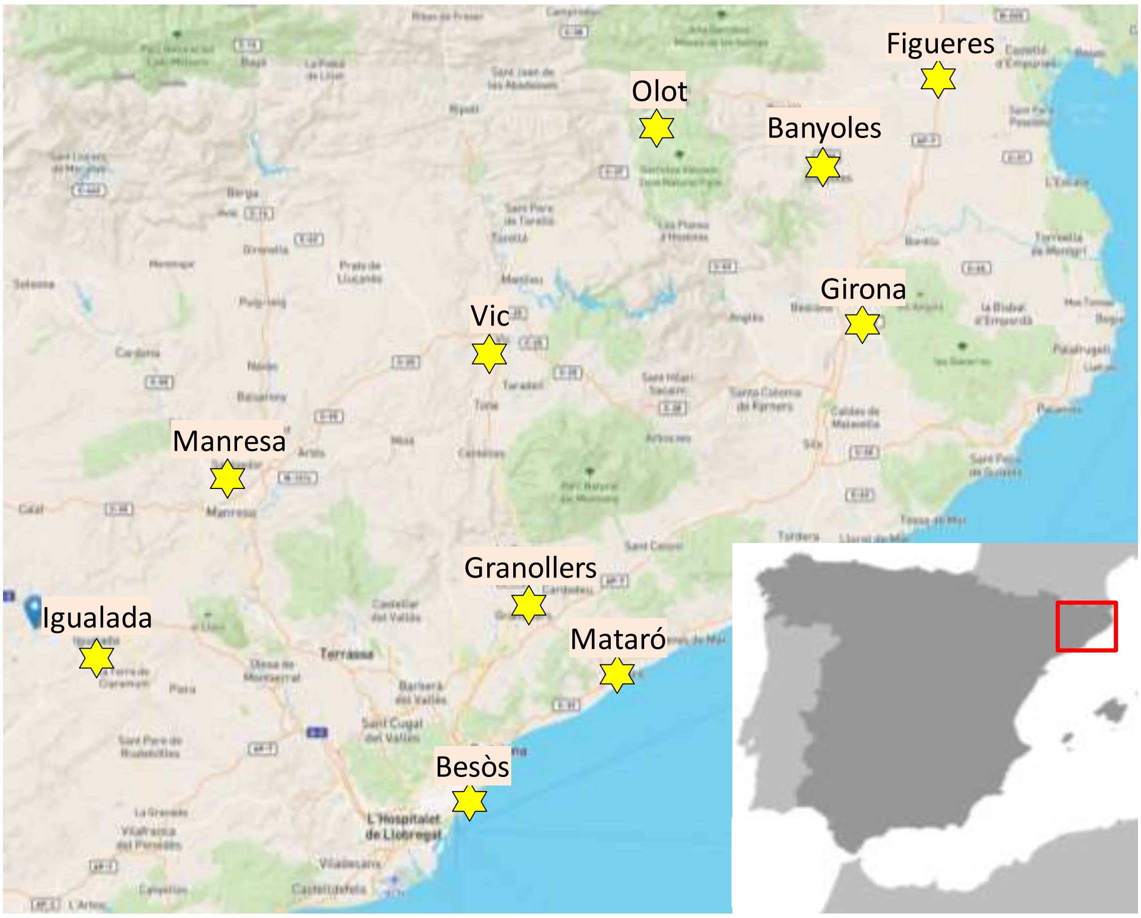

Figure S1.- Location of the 10 WWTPs where samples were collected.

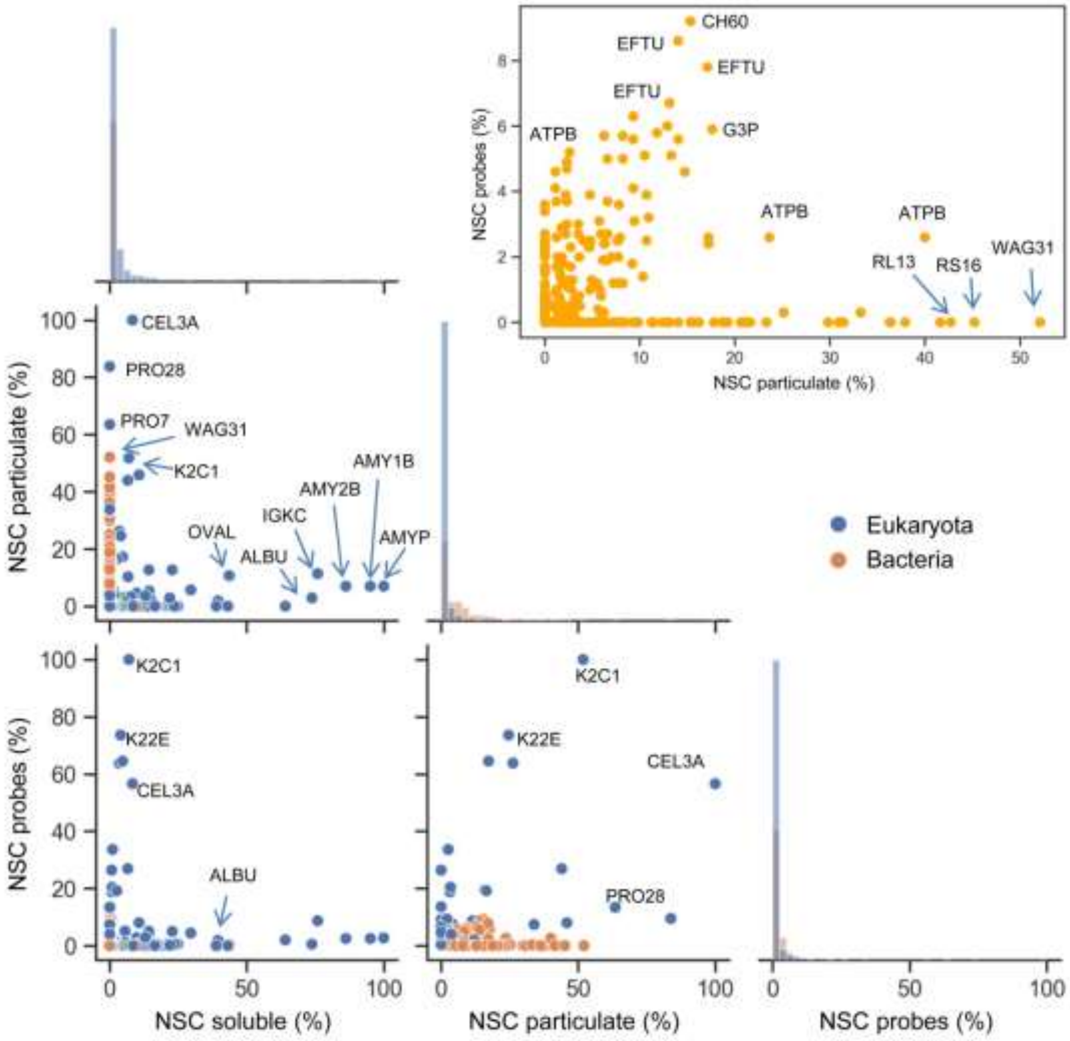

Figure S2.- Comparison of the Eukaryote (blue) and Bacterial (orange) proteins in the wastewater soluble and particulate fractions and those found bound to the polymeric probes described in a previous work [23]. Insert: details on the bacterial proteins. Abundance estimation (#NSC) was normalized to the most abundant component in each fraction. Diagonals are the histograms of the protein distribution in each fraction (soluble, particulate, and probes, from top-left to bottom right).

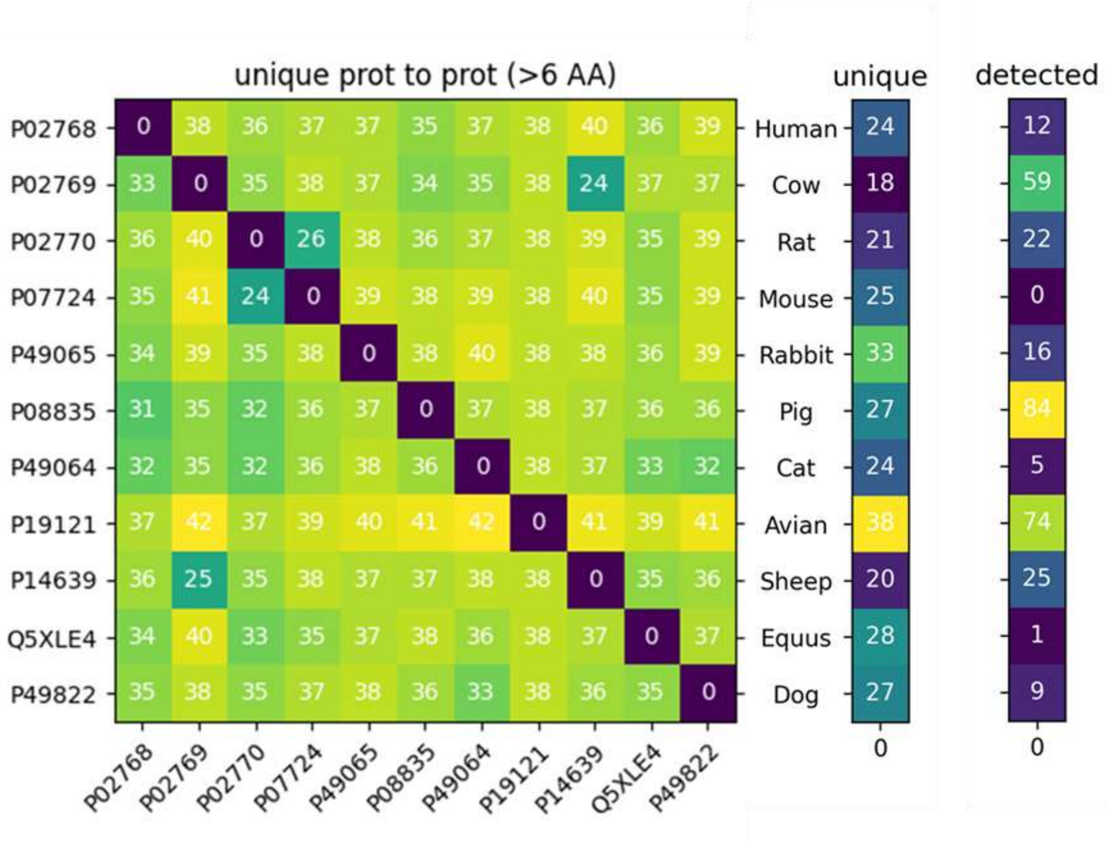

Figure S3.- Number of tryptic sequences that are different between any pair of the represented albumins (left), that are different from any other (unique in this albumin set) (center,) and unique sequences that were detected in our samples (right). Calculation of unique sequences considers only canonical tryptic peptides, whereas the experimental data includes sequences with missed cleavages.

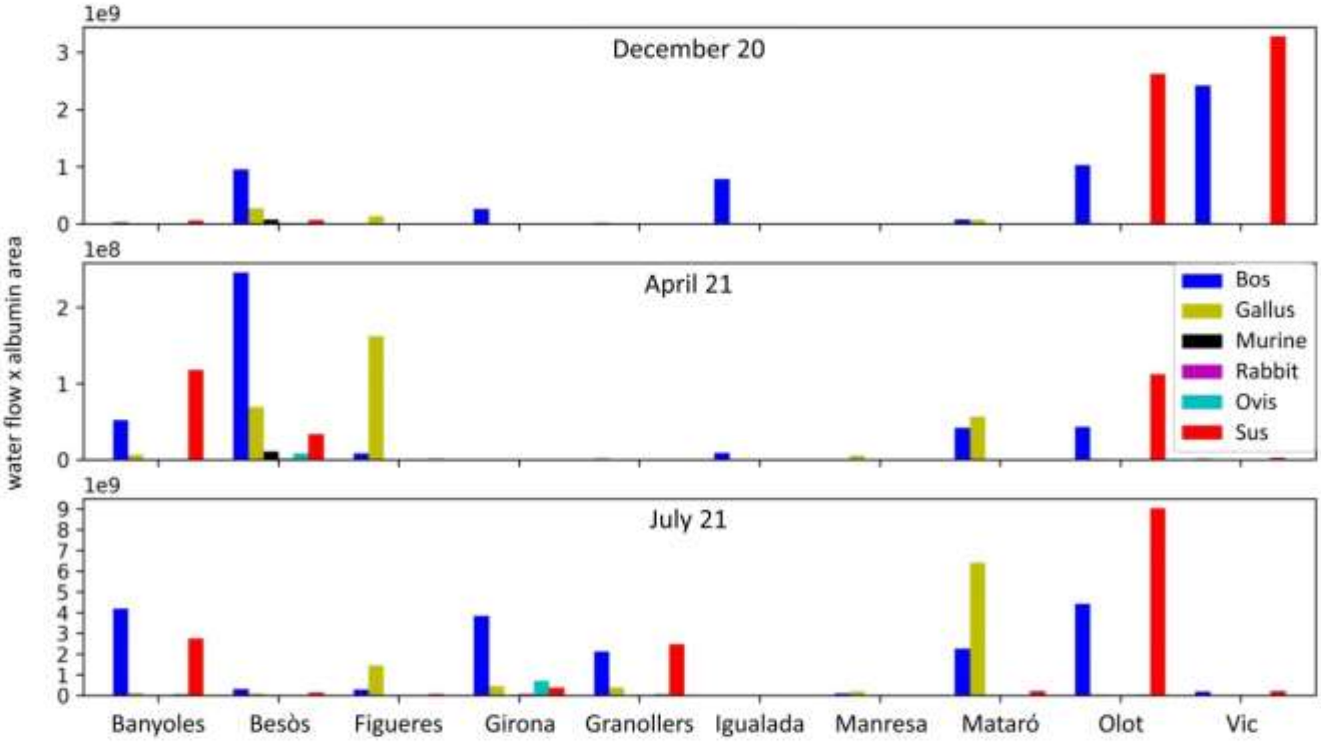

Figure S4.- Albumin profiles from farm animals in the three campaigns.

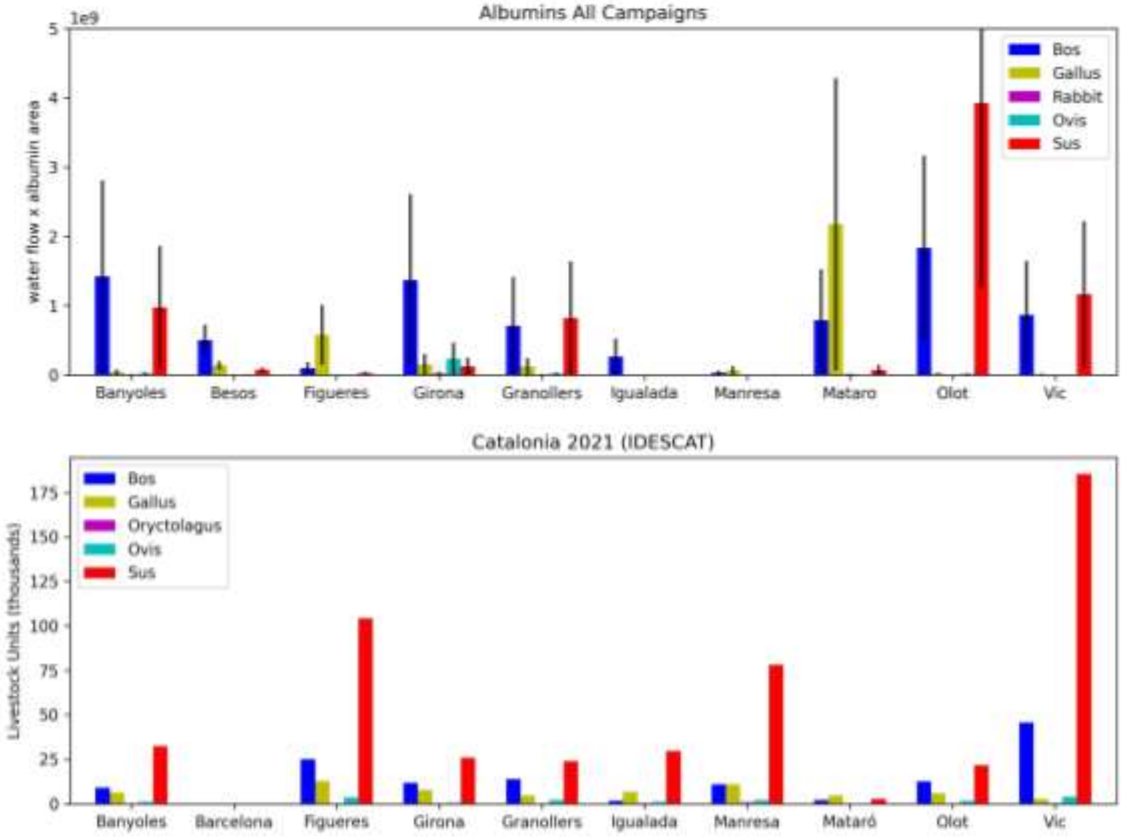

Figure S5.- Comparison of the average albumin profiles with the livestock units in the county in which the WWTP is located (IDESCAT data for 2021, https://www.idescat.cat/).

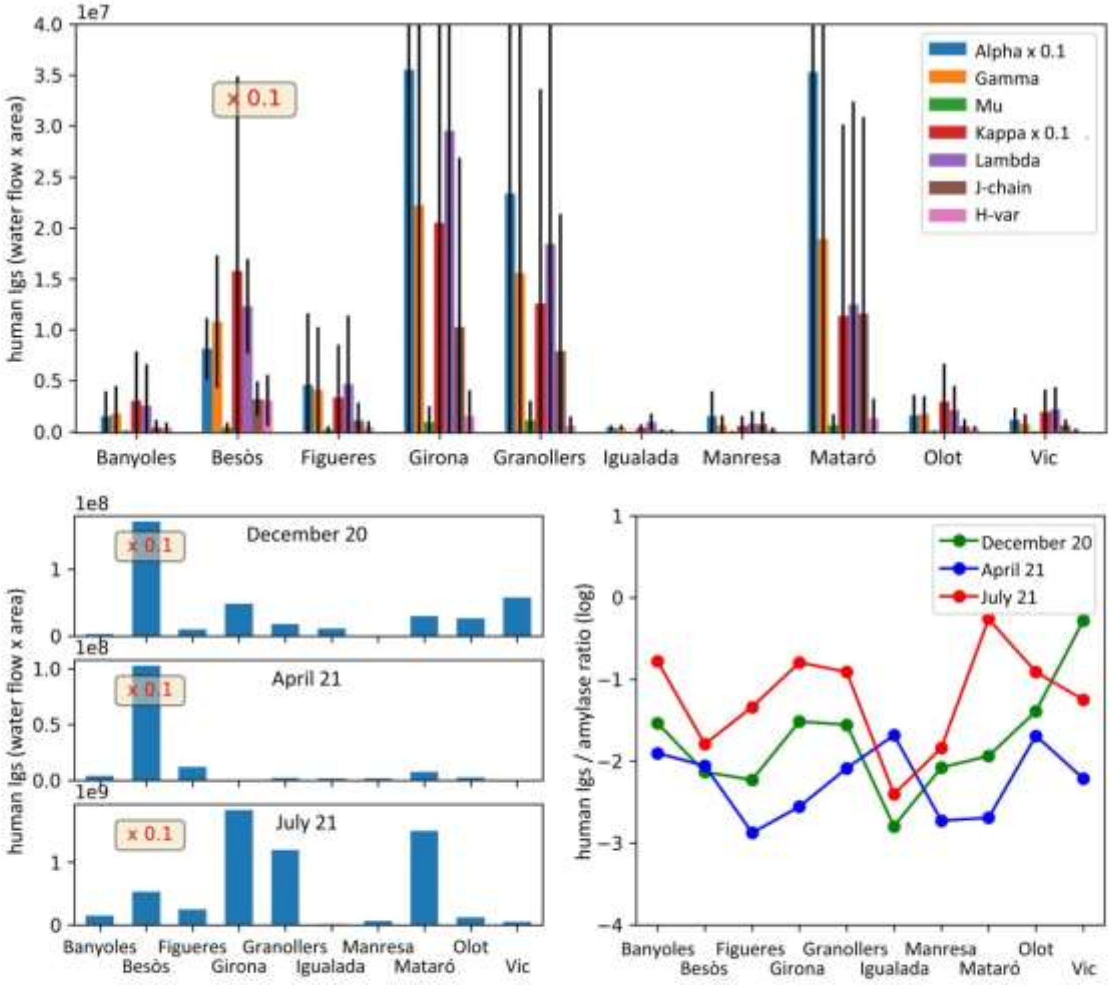

Figure S6.- Distribution of human Igs in wastewater from the different municipalities (top), total human Igs abundance per site and campaign (bottom left), and human Ig/amylase ratios at the different sites and campaigns (bottom, right).

Table S1.- Collection sites. Population equivalent, population served, and water treated at the different WWTPs.

WWTP	Population (thousands)		Water treated[1] (m^3/d)
	Equivalent[1]	Served[2]	
Banyoles	53	28	12,000
Besòs	2,844	1,502	525,000
Figueres	111	53	17,000
Girona	206	159	55,000
Granollers	122	100	30,000
Igualada	286	67	20,000
Manresa	196	93	53,500
Mataró	451	190	57,000
Olot	99	46	17,000
Vic	340	55	25,000

[1] *Agència Catalana de l'Aigua (21/11/2022), https://aca.gencat.cat/ca/laigua/infraestructures/estacions-depuradores-daigua-residual/*

[2] *https://sarsaigua.icra.cat/ and https://www.epdata.es/*

Table S2.- Species represented by at least two proteins in the set of proteins selected for semiquantitative analysis.

Species	Proteins
Homo sapiens	169
Sus scrofa	59
Bos taurus	55
Gallus gallus	37
Rattus norvegicus	9
Mus musculus	9
Triticum aestivum	8
Oryctolagus cuniculus	7
Canis lupus familiaris	7
Solanum tuberosum	5
Ovis aries	4
Pseudomonas aeruginosa	4
Hordeum vulgare	3
Capra hircus	3
Felis catus	2
Prunus dulcis	2
Bacteroides vulgatus	2
Bacillus amyloliquefaciens	2
Lachnospira eligens	2
Equus caballus	2
Danio rerio	2
Clostridioides difficile	2
Pongo abelii	2
Malus domestica	2
Solanum lycopersicum	2
Dictyostelium discoideum	2

Table S3.- Wastewater-origin discriminant proteins used for LDA-supervised classification

Accession	Name	Species	Gene
P02769	Albumin	*Bos taurus*	ALB
P0DUB6	Alpha-amylase 1A	*Homo sapiens*	AMY1A
P08835	Albumin	*Sus scrofa*	ALB
P19121	Albumin	*Gallus gallus*	ALB
P02768	Albumin	*Homo sapiens*	ALB
P01012	Ovalbumin	*Gallus gallus*	SERPINB14
P09571	Serotransferrin	*Sus scrofa*	TF
P00689	Pancreatic alpha-amylase	*Rattus norvegicus*	Amy2
P01009	Alpha-1-antitrypsin	*Homo sapiens*	SERPINA1
P02789	Ovotransferrin	*Gallus gallus*	TF
Q29443	Serotransferrin	*Bos taurus*	TF
P02787	Serotransferrin	*Homo sapiens*	TF
Q9TTE1	Serpin A3-1	*Bos taurus*	SERPINA3-1
P14639	Albumin	*Ovis aries*	ALB
P49064	Albumin	*Felis catus*	ALB
P49065	Albumin	*Oryctolagus cuniculus*	ALB
P50447	Alpha-1-antitrypsin	*Sus scrofa*	SERPINA1
P49822	Albumin	*Canis lupus familiaris*	ALB
P15693	Intestinal-type alkaline phosphatase 1	*Rattus norvegicus*	Alpi
P34955	Alpha-1-antiproteinase	*Bos taurus*	SERPINA1
P27425	Serotransferrin	*Equus caballus*	TF
P12725	Alpha-1-antiproteinase	*Ovis aries*	SERPINA1
A6YF56	Albumin	*Mesocricetus auratus*	ALB
Q5XLE4	Albumin	*Equus asinus*	ALB
